# Splice variants and promoter methylation status of the Bovine *Vasa* Homology (*Bvh*) gene may be involved in bull spermatogenesis

**DOI:** 10.1186/1471-2156-14-58

**Published:** 2013-07-01

**Authors:** Hua Luo, Yang Zhou, Yingxia Li, Qifa Li

**Affiliations:** 1 College of Animal Science and Technology, Nanjing Agricultural University, Nanjing 210095, P.R. China

## Abstract

**Background:**

Vasa is a member of the DEAD-box protein family that plays an indispensable role in mammalian spermatogenesis, particularly during meiosis. Bovine vasa homology (*Bvh*) of *Bos taurus* has been reported, however, its function in bovine testicular tissue remains obscure. This study aimed to reveal the functions of *Bvh* and to determine whether *Bvh* is a candidate gene in the regulation of spermatogenesis in bovine, and to illustrate whether its transcription is regulated by alternative splicing and DNA methylation.

**Results:**

Here we report the molecular characterization, alternative splicing pattern, expression and promoter methylation status of *Bvh*. The full-length coding region of *Bvh* was 2190 bp, which encodes a 729 amino acid (aa) protein containing nine consensus regions of the DEAD box protein family. *Bvh* is expressed only in the ovary and testis of adult cattle. Two splice variants were identified and termed *Bvh*-*V4* (2112 bp and 703 aa) and *Bvh*-*V45* (2040 bp and 679 aa). In male cattle, full-length *Bvh* (*Bvh*-*FL*), *Bvh*-*V4* and *Bvh*-*V45* are exclusively expressed in the testes in the ratio of 2.2:1.6:1, respectively. Real-time PCR revealed significantly reduced mRNA expression of *Bvh*-*FL*, *Bvh*-*V4* and *Bvh*-*V45* in testes of cattle-yak hybrids, with meiotic arrest compared with cattle and yaks with normal spermatogenesis (P < 0.01). The promoter methylation level of *Bvh* in the testes of cattle-yak hybrids was significantly greater than in cattle and yaks (P < 0.01).

**Conclusion:**

In the present study, *Bvh* was isolated and characterized. These data suggest that Bvh functions in bovine spermatogenesis, and that transcription of the gene in testes were regulated by alternative splice and promoter methylation.

## Background

Vasa is an important member of the DEAD (Asp-Glu-Ala-Asp) box family, which were first discovered in Drosophila [1,2]. In mammals, Vasa is only expressed in the germline, and is widely used as a molecular marker for the study of gametogenesis and the origin, migration and differentiation of primordial germ cells (PGCs) [3,4]. In the adult testis, the expression of *Vasa* occurs before meiosis, and continues until post-meiotic stage. *Vasa* is expressed abundantly in spermatogonia and spermatocytes that have not yet entered the first meiotic division, but is expressed at a low level in early germ cells, and not at all in later stage germ cells, spermatozoa and somatic cells. Vasa is an essential protein for spermatogenesis [5-7]. *Vasa* mutations cause defects in PGC differentiation and amplification. *Mvh* (mouse vasa homology) mutations cause germ cell apoptosis resulting from incomplete meiosis, ultimately leading to a lack of sperm production and male sterility [8,9]. Spermatogenesis was blocked in *Mvh* knockout mice, which led to male sterility; however, *Mvh* knockout females were fertile. In *Mvh* knockout homozygous mutant mice, spermatogenesis was blocked at zygotene of the first meiotic prophase, which led to apoptosis and lack of sperm production. Therefore, in mouse spermatogenesis, the successful completion of zygotene depends on the expression of *Mvh*[10].

The hybridization between two different species frequently results in reproductive isolation [11], for example for between horse (*Equus caballus*) and donkey (*Equus asinus*) [12], and cattle (*Bos taurus*) and yak (*Bos grunniens*) [13]. The cattle-yak is an interspecific hybrid offspring of cattle and yaks, and reproductive isolation results from the male sterility in the F1 hybrid [14,15]. The cattle-yak hybrid shows strong heterosis compared with cattle and yaks, and the cattle-yak hybrid can significantly improve the production performance of yaks [16]; however, the male sterility in the F1 hybrid is a major obstacle to yak crossbreeding and exploitation of heterosis [17]. Determining the mechanism of male sterility in cattle-yaks has both theoretical significance and practical value for research on reproductive isolation of interspecific hybrids, species formation and exploitation of heterosis. The male sterility of cattle-yak hybrids in the F1 generations is caused by spermatocyte meiosis arrest [13], and the phenotype of the spermatogenesis blockage is similar to the phenotype of *Mvh* knockout mice [10].

Recently, some studies on the bovine *Vasa* homolog (*Bvh*) were reported [18], however, its molecular and evolutionary feature, and function in bovine testicular tissue remains obscure. The present study aimed to identify and characterize the bovine *Vasa* homolog (*Bvh*) of the cattle, yaks and their interspecific hybrid cattle-yaks. We also aimed to use the cattle-yak as the model of male sterility to investigate the expression distribution, forms of splice variant and status of promoter methylation of the *Bvh* gene among cattle, yak and cattle-yak hybrid to assess the role of *Bvh* in bovine spermatogenesis and its regulation.

## Results

### Identification and characterization of the Bvh gene

**Figure 1:**
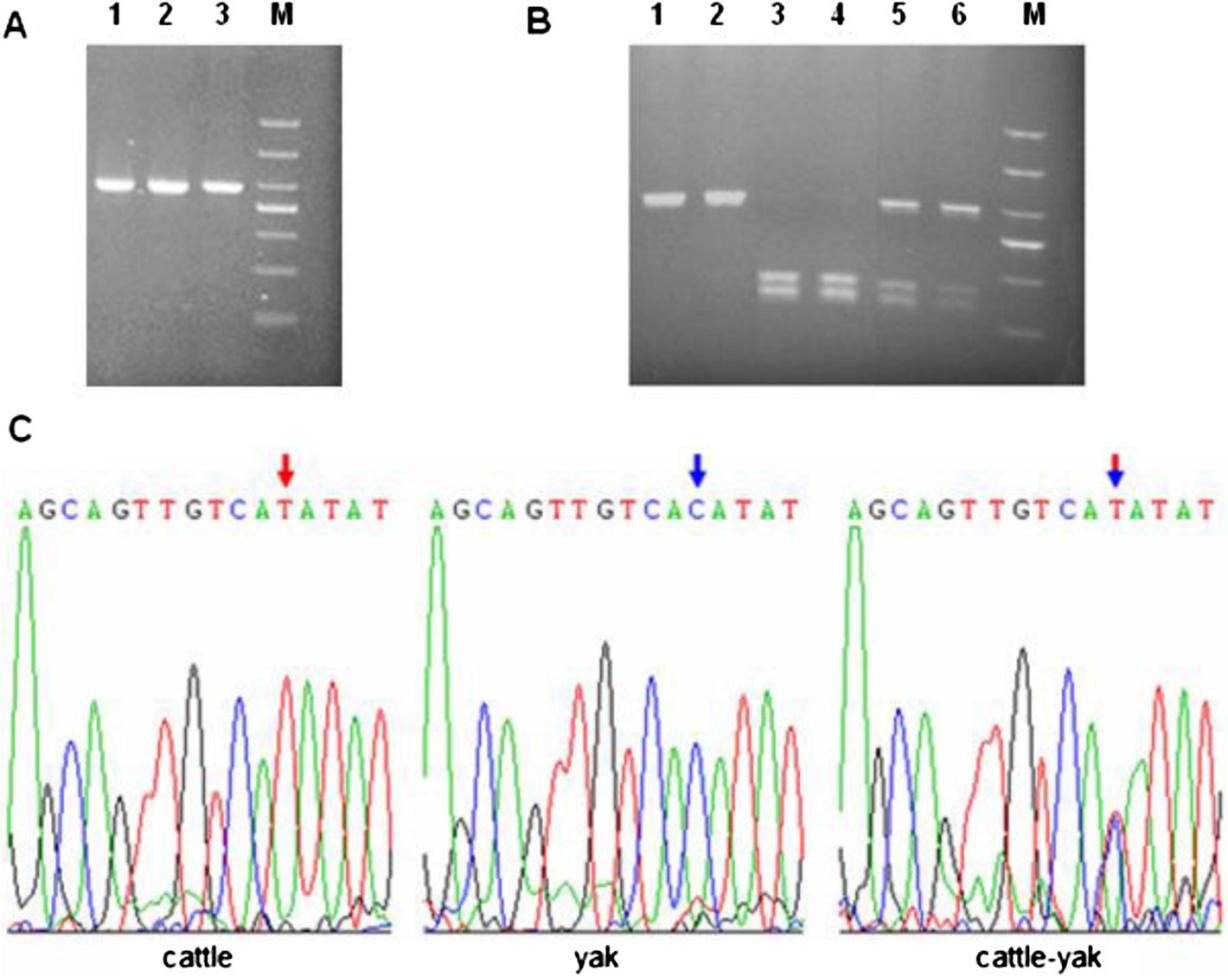
**PCR**-**RFLP assay of *Bvh *nt1202 site. A**. Gel photograph of 337 bp fragments of *Bvh*. Line1-3 were cattle, yak and cattle-yak hybrid, respectively. Molecular marker was DL500. **B**. Restriction patterns of 337 bp fragments of *Bvh* digesting with *Nde* I enzyme on 1.5% agarose gel. Line 1 and 2 was genotype TT (337 bp). Line 3 and 4 was genotype CC (232 bp + 105 bp 337 bp). Line 5 and 6 was genotype TC (337 bp + 232 bp + 105 bp). **C**. Sequence of different genotypes at the nt1202 of Bvh gene. Arrow denotes substitution position. Red arrow denotes genotype TT, Blue arrow denotes genotype CC, Red and blue arrow denotes genotype TC.

The full length coding region of *Bvh* from cattle, yaks and cattle-yak hybrids were all 2190 bp (GenBank accession no. JX437185, JX437186 and JX437187, respectively). The coding region of cattle *Bvh* was 100% homologous to that of the cattle-yak hybrid, and 99.95% homologous to the yak sequence, with only one nonsynonymous substitution (T→C) detected at nt1202, causing an amino acid change (Ile401Thr). The *Bvh* nt1202T>C polymorphism was determined in 231 individuals of the three populations (cattle, yaks and cattle-yak hybrids) using a PCR-RFLP assay with *Nde* I enzyme and sequencing. The result showed that the genotype TT was detected only in the cattle population, CC only in the yak population, and TC in the cattle-yak hybrid population (Figure 1).

**Figure 2:**
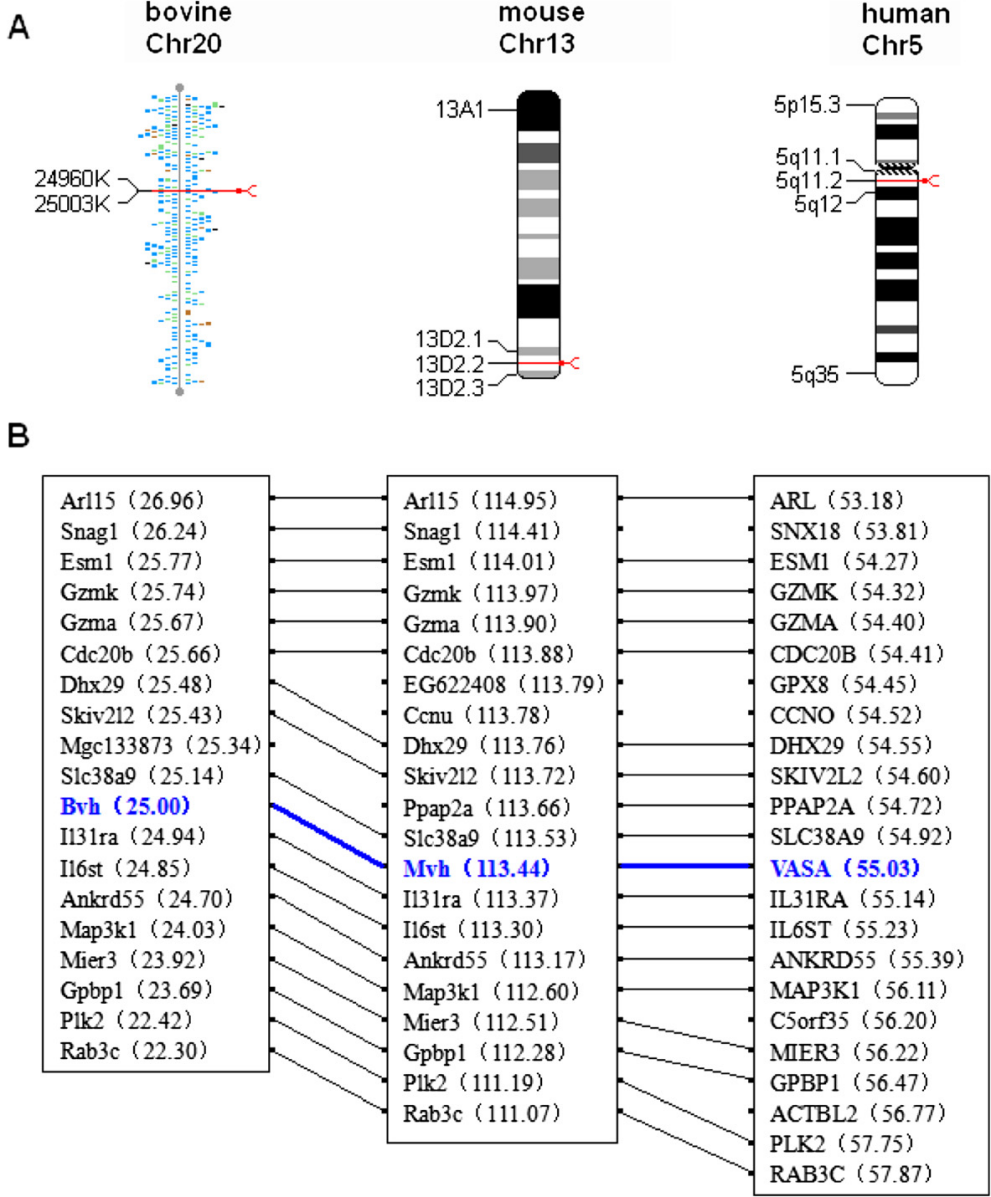
**Chromosomal location and Chromosomal synteny of bovine**, **mouse and human vasa gene. A**. Chromosomal location of vasa gene. **B**. Chromosomal synteny of vasa gene. Chr, chromosome. Numericals in parentheses are chromosomal positions.

The nucleotide sequence of the coding region of cattle *Bvh* was very similar to those of the human (91.84%), mouse (87.80%) and dog (86.85%), but not very similar to *Bvh* of the chicken (58.20%). Comparing the *Bvh* cDNA sequence with the bovine genomic sequence showed that the genomic sequence of *Bvh* consisted of 17 exons and 16 introns. *Bvh* was mapped to a position within NW_001493943 on chromosome 20 (Figure 2A) by electronic chromosomal localization analysis. To further determine whether *Bvh* was the evolutionary ortholog of human *Vasa* and mouse *Mvh*, we analyzed their chromosomal syntenic relationships. The *Bvh*-bearing region contains 21 genes, including *Bvh* and exhibits a conserved synteny to the *VASA*-containing region on human chromosome 5 and *Mvh*-containing region on mouse chromosome 13 (Figure 2B).

**Figure 3:**
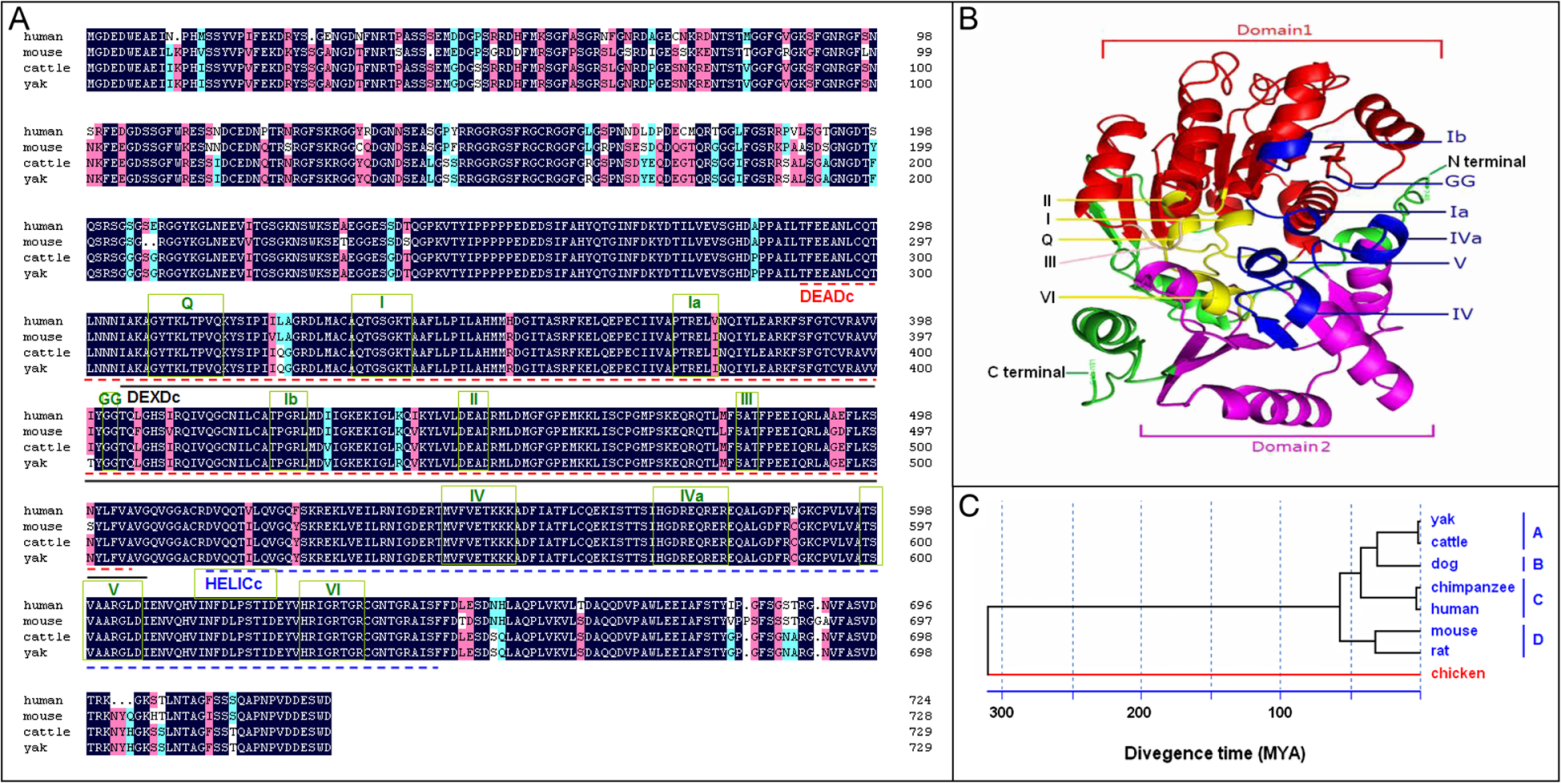
**Identification of *Bvh*. A**. Alignment of Bvh protein with human (NP_077726) and mouse (NP_001139357). Black boxes indicate positions at which the residues are identical and grey boxes highlight residues that are similar. Functional domains and motifs are indicated in underline and pane, respectively. **B**. The three-dimensional structure of the helicase core domains of the *Bvh* protein. The conserved sequence motifs within these domains are shown, with colour coding that corresponds to the primary function of the domain. Red indicate Domain 1 (DEADc domain). Purple indicate Domain 2 (HELICc domain). Green indicate unconcerved region. Yellow indicate ATP binding site. Blue indicate RNA binding site. Pink indicate communication between ATP-binding and RNA-binding sites. **C**. The phylogenetic relationship of bovine with other mammals and their divergence time. Mammal groups in blue and bird groups in red. Drawings are based on Graves and Westerman [21], and Xu et al [22].

*Bvh* encodes a protein of 729 amino acids (aa) with molecular weight of 79.48 kDa (cattle) and 79.47 kDa (yaks). The cattle Bvh protein sequence is 89.88% and 91.08% identical in the mouse Mvh and human VASA sequences, respectively. The mouse and human are 88.37% identical to each other, but only 52.54% identical to the chicken Cvh protein, indicating that the amino acid sequence of vasa is extensively conserved in mammals. *In silico* subcellular localization analysis predicted that the Bvh protein would be localized to the cytoplasm, which was consistent with the results for human [19] and mouse [20]. Further analysis indicated that Bvh contains three conserved domains DEADc (from Thr292 to Ala506), DEXDc (from Ile305 to Gly508) and HELICc (from Asp516 to Phe645). The amino acid sequences within these regions are more conserved than the N or C terminal regions (Figure 3A). In addition, seven conserved motifs (Q, I, Ia, Ib, GG, II and III) were identified in Domain 1 (DEADc domain), and four motifs (IV, Va, V and VI) in Domain 2 (HELICc domain). The amino acid sequence, constitution, arrangements and location of functional domains and motifs of Bvh are very similar to the Vasa protein from other mammalian species, which indicated that Bvh is a member of the DEAD-box protein family with ATP-dependent RNA helicase activity.

The three-dimensional structure of Bvh Domain 1 and Domain 2 are represented in Figure 3B. Domain 1 (DEADc domain) consists of 10 *α*-helices and seven *β*-sheets in the order *αααβαβαβαβαβααβαβ*. The seven *β*-sheets are all arranged in parallel in the internal, *α*-helix arranged along to the parallel of a week in external. Domain 2 (HELICc domain) consists of five *α*-helices and six *β*-sheets in the order *βαβαβαβαβαβαβ*, which is similar to the structure of Domain 1. The structures of Domain 1 and Domain 2 are both similar to recombinase A (RecA), obvious fissures are formed between the two recombinase A (RecA-like) domains, which are associated with binding to nucleic acids. In addition, there are two *α*-helices and a *β*-sheet in the N-terminus of the two conserved extraterritorial regions near Domain 1, which formed a banded structure around the fissure between the two domains. There was also a longer spiral in the C terminal sequence near Domain 2, which surrounded the *β*-sheet with the *α*-helices of Domain 2 in an almost parallel arrangement. In addition, ATP binding and hydrolysis-related motifs Q-I, II, and VI form the fissure within the side of the protein, related to RNA nucleic acid binding motifs of Ia, Ib, GG, III, IV, IVa, V on the opposite side of the protein and almost perpendicular to the fissure, in the surface of the two motifs. Motif III is the DEAD box, motifs II interacts with Mg^+ +^, motif I is the ATP binding site, and VI is related to the transition state stability of the protein [23].

### Molecular evolution of Vasa in mammals

Four hundred and twenty six polymorphic sites were detected in nucleotide sequence of the *Vasa* coding region among seven mammal species (human, chimpanzee, mouse, rat, dog, cattle and yak), and the nucleotide diversity (Pi) was 0.0850. Among the base substitutions, there were 122 transitions and 57 transversions, and the transition/transversion ratio (Ts/Tv, R) was 2.13, which was significantly greater than the critical value (2.0) of Ts/Tv, indicating that *Vasa* possesses a strong transition preference. Neutrality tests found that Tajima’s D value for *Vasa* in mammals was −0.18086 (Not significant, P > 0.10), which demonstrated that the polymorphism frequency of *Vasa* in is low in mammals. Analysis of the nonsynonymous substitution (d_N_) and synonymous substitutions rate (d_S_) found that the value of d_N_ was 0.062 (standard error SE = 0.006), d_S_ was 0.240 (SE = 0.026) and d_N_/d_S_ was significantly less than 1 (Z test, P <0.01), which indicated that the evolution of mammalian *Vasa* genes has been influenced by purifying selection (negative selection). Thus, the *Vasa* gene is relatively conserved in different mammal species.

A phylogenetic tree of seven mammals was constructed according to the amino acid sequences of Vasa proteins. The tree showed that the outgroup (chicken, birds) was clustered alone, whereas the seven mammals clustered together (Figure 3C). The seven species of mammals were divided into four obvious clades. Yak and cattle, which belongs to *Bovinae*, were clustered initially as clade A, domestic dog (*Canine*) was clustered as clade B, human and chimpanzee (*Hominidae*) were clustered as clade C, and the mouse and rat (*Murine*) were clustered as clade D. This is consistent with the traditional classification. Previous data showed that mammals and birds diverged about 310 million years ago (MYA) [21,24]. Therefore, according to the divergence time of mammals and birds and the amino acid sequence divergence of the Bvh protein, Figure 3C indicates that yaks and cattle diverged about 0.51 MYA, which is consistent with the results of Ritz et al. [25], MacEachern et al. [26] and Xie et al. [27], whose results were based on microsatellite markers, 15 autosomal genes and the mitochondrial cytochrome b gene, respectively.

### Characterization of alternatively spliced Bvh transcript variants

**Figure 4:**
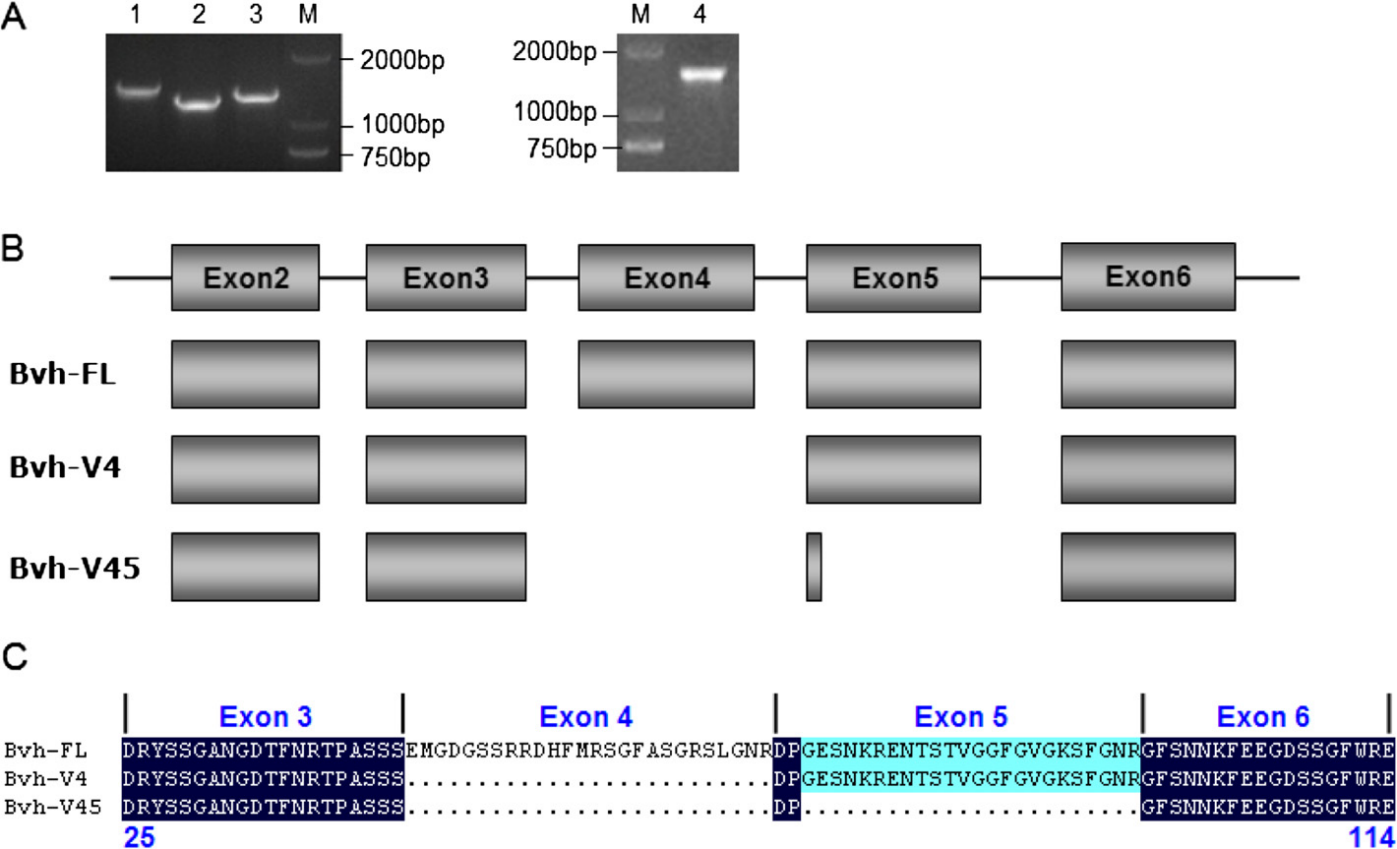
**Alternative splice of *Bvh *in bovine testes. A**. Agarose gel electropherograms of the splice variants from testis tissues developed by RT-PCR. Line 1-3 were amplification fragment of primer 1, line1 was full-length transcript, and line 2-3 were splice variants. Line 4 was amplification fragment of primer 2. Molecular marker was DL2000. **B**. Splice variants in coding region of *Bvh*. Number labelled boxes indicate the genomic regions identical to cDNA (exon2 to 6). FL means the full-length transcript, V4 is splice variant lack of exon4 and V45 is splice variant lack of exon4 and 5. **C**. Amino acids changes caused by alternative splicing. The alignment figures display the changes at the protein level of two identified splice variants compared to full-length protein.

Previous reports demonstrated that *Vasa* transcripts are present as different splice isoforms in several animals, such as zebrafish [28], sea urchin [29], flatworm [30], clam worm [31], tammar wallaby and platypus [2]. However, alternative splicing of *Vasa* in human, mouse and domestic animals has not been reported. To determine whether the cattle *Bvh* also undergoes alternative splicing, RT-PCR was performed with primers P1 and P2 (Table 1), which cover the complete coding sequences, and pooled cDNA prepared from testis. Thirty clones were collected from the RT-PCR products and sequenced. The result showed that two splice variants were identified within the amplification fragments for primers P1, but no alternatively spliced isoforms were identified within the amplification fragment for primers P2 (Figure 4A).

**Table 1:** Primers in this study

	**Gene**	**GenBank ID**	**Primer sequence (5′-3′)**	**Annealing temp (°C)**	**Product size (bp)**	**Application**
P1	*Bvh*	AF541971	F: GAAGATTGGGAAGCAGAAA	58	1457	cDNA clone
			R: CTGACGCTGTTCCTTTGAT			
P2	*Bvh*	AF541971	F: AACAGGCATAAACTTTGACA	59	1525	cDNA clone
			R: GGGTGGGAGTAAGAACAGA			
P3	*Bvh*-*FL*	AF541971	F: TGCCTCTGGGAGGAGTTTGG	60	294	Real-time-PCR
			R: GGCAACCTCGGAAACTACC			
P4	*β*-*actin*	NM_173979	F: CGGACTGTTAGCTGCGTTAC	60	164	Real-time PCR
			R: CACCTTCACCGTTCCAGTT			
P5	*Bvh*	JX437185	F: AAG GTA AGA ATC TCC CAC TC	58	337	PCR-RFLP
			R: GTC CTT GGC ACT TTC TAC AC			
P6	*Bvh*-*V4*	JX437188	F: AGATCCTGGTTTTTCAAATAAC	60	193	Real-time PCR
			R: GCAACCTCGGAAACTACCT			
P7	*Bvh*-*V45*	JX437189	F: CGTCAGATCCTGGTGAGTCT	60	83	Real-time PCR
			R: TCTGTTTCCAAAACTCTTT			
P8	*Bvh*	NW_003104511	F: GGATTGTAGTAGGTAAAAAAAGGAGA	53	346	Bisulfite sequencing PCR
			R: TCCAACAACAAATAACACCAA			

Among the 30 clones generated using primer P1, 14 clones (46.67%) are full-length cattle *Bvh* cDNAs (*Bvh*-*FL*), 11 (36.67%) lack 78 bp from complete exon4 (*Bvh*-*V4*, GenBank accession no. JX437188), and five (16.67%) lack 150 bp from complete exon4 and partial exon5 (*Bvh*-*V45*, GenBank accession no. JX437189) (Figure 4B). These results suggest that *Bvh*-*FL* and splice variant *Bvh*-*V4* are the major isoforms in cattle testes, but the *Bvh*-*V45* splice variant isoform is relatively rare. Further analysis found that splicing of *Bvh*-*V4* and *Bvh*-*V45* follow the “GT-AG” splicing rule. Compared with the full-length Bvh protein (Bvh-FL), the proteins encoded by *Bvh*-*V4* and *Bvh*-*V45* lack 26 aa and 50 aa respectively (Figure 4C). The nucleotides lost are 3 N; therefore, neither splice variation causes a frameshift mutation or early termination of translation. The splice sites of two splice variants are both located outside the region encoding the conserved domain of the DEAD-box family (Domain 1 and 2) in the N-terminus and the missing bases are all located 5′ to the conserved region. Thus, Bvh-V4 and Bvh-V45 both contain the conserved domains and functional motifs of DEAD-box family, and the four RGG sequence and four GG sequence related to RNA-binding in the N-terminus. The results showed that the alternative splicing does not affect the overall protein structure, suggesting that Bvh-V4 and Bvh-V45 retain the fundamental biological functions of the Bvh protein.

### Expression analysis of Bvh and its splice variants

**Figure 5:**
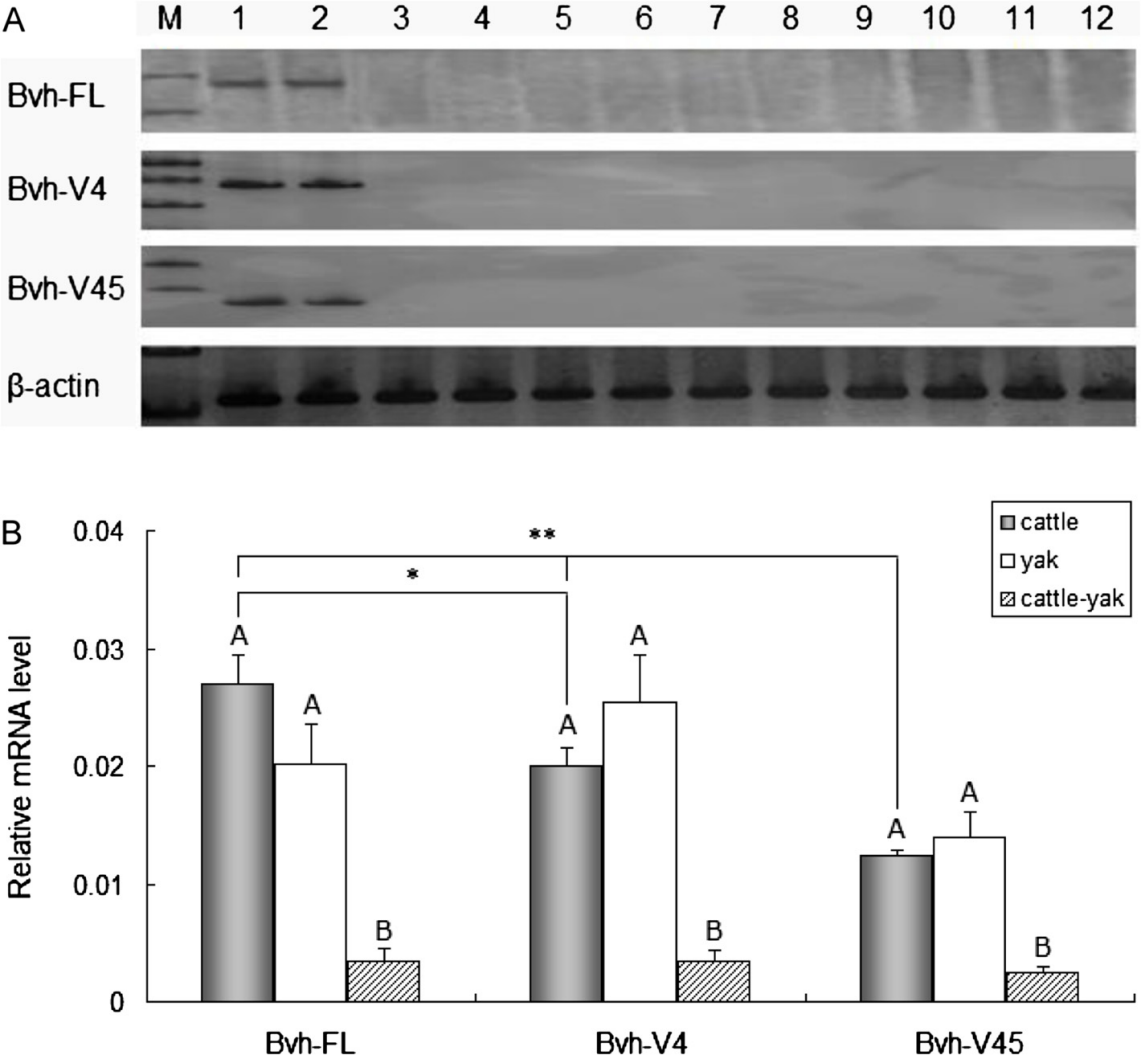
**The expression of *Bvh *gene and its splice variants. A**. The expression profile of *Bvh* and its splice variants in cattle, yak and cattle-yak hybrid. DNA marker (M) was DL100. Line1-12 were testis, ovary, epididymis, glandula accessoria, hypophysis, hypothalamus, heart, liver, spleen, kidney, lung and muscle, respectively. **B**. The mRNA expression levels of *Bvh* gene and its splice variants in cattle, yak and cattle-yak hybrid. Different uppercase letters represent the difference of expression levels among populations are significant (P < 0.01). ^*^ indicate the significantly difference (P < 0.05), ^**^ indicate the significantly difference (P < 0.01).

To assess the mRNA expression patterns of *Bvh*, RT-PCR was performed using the P3 primers described in Table 1. PCR products for *Bvh*-*FL* were only detected in the testis and ovary tissues of adult cattle, and not detected in the epididymis, glandula accessoria, hypophysis, hypothalamus, heart, liver, spleen, kidney, lung and muscle, which indicated that *Bvh* is a testis- and ovary-specific expressed gene (Figure 5A). The mRNA expression patterns of two splice variants *Bvh*-*V4* and *Bvh*-*V45* were consistent with *Bvh*-*FL*.

RT–PCR analysis showed distinct signal intensities for *Bvh*-*FL*, *Bvh*-*V4* and *Bvh*-*V45* in the testes of adult cattle (Figure 5A). To accurately estimate their relative proportions in the testes of adult cattle, real-time PCR using the primers P3, P6 and P7 (Table 1) was performed. The result showed that *Bvh*-*FL* was the most abundantly expressed, followed by *Bvh*-*V4* and *Bvh*-*V45*. The expression level of *Bvh*-*FL* was significantly higher than that of *Bvh*-*V4* (P < 0.05), and the expression levels of *Bvh*-*FL* and *Bvh*-*V4* were significantly higher than that of *Bvh*-*V45* (P < 0.01) (Figure 5B). The relative ratio for *Bvh-FL: Bvh-V4: Bvh-V45* was 2.2:1.6:1.

To estimate whether the expression of *Bvh* and the splice variants was correlated with hybrid male sterility, we determined the mRNA expression levels of *Bvh*-*FL*, *Bvh*-*V4* and *Bvh*-*V45* in the testes of cattle and yaks with normal spermatogenesis, and their interspecific hybrid cattle-yak with male sterility. Real-time PCR revealed significantly reduced mRNA expression of *Bvh*-*FL*, *Bvh*-*V4* and *Bvh*-*V45* in the testes of cattle-yak hybrids compared with cattle and yaks (P < 0.00001); however, no significant difference was observed between cattle and yaks (P > 0.05) (Figure 5B). The decreases were 6 to 8 -, 6 to 7 - and 5 to 6 - fold for *Bvh*-*FL*, *Bvh*-*V4* and *Bvh*-*V45*, respectively.

### Promoter methylation

Based on the coding sequence of cattle, we retrieved 8 Kb of the 5′ flanking region sequence of *Bvh* (−7927 ~ +73, relative to the initiation codon, ATG, at +1) (nt5082427-5090426, in NW_003104511.1) from the cattle genome database, which included the promoter, exon1, intron1 and exon2. The core promoter region was determined as -1449nt to -1199nt, which was 251 bp in length and included transcription factor (TF) binding sites, such as those for Sp1, T-Ag, AP-2, UCE.2 and INF.1. A predicted CpG island was identified at -1547nt to -630nt (918 nucleotides), which included the predicted core promoter region.

**Figure 6:**
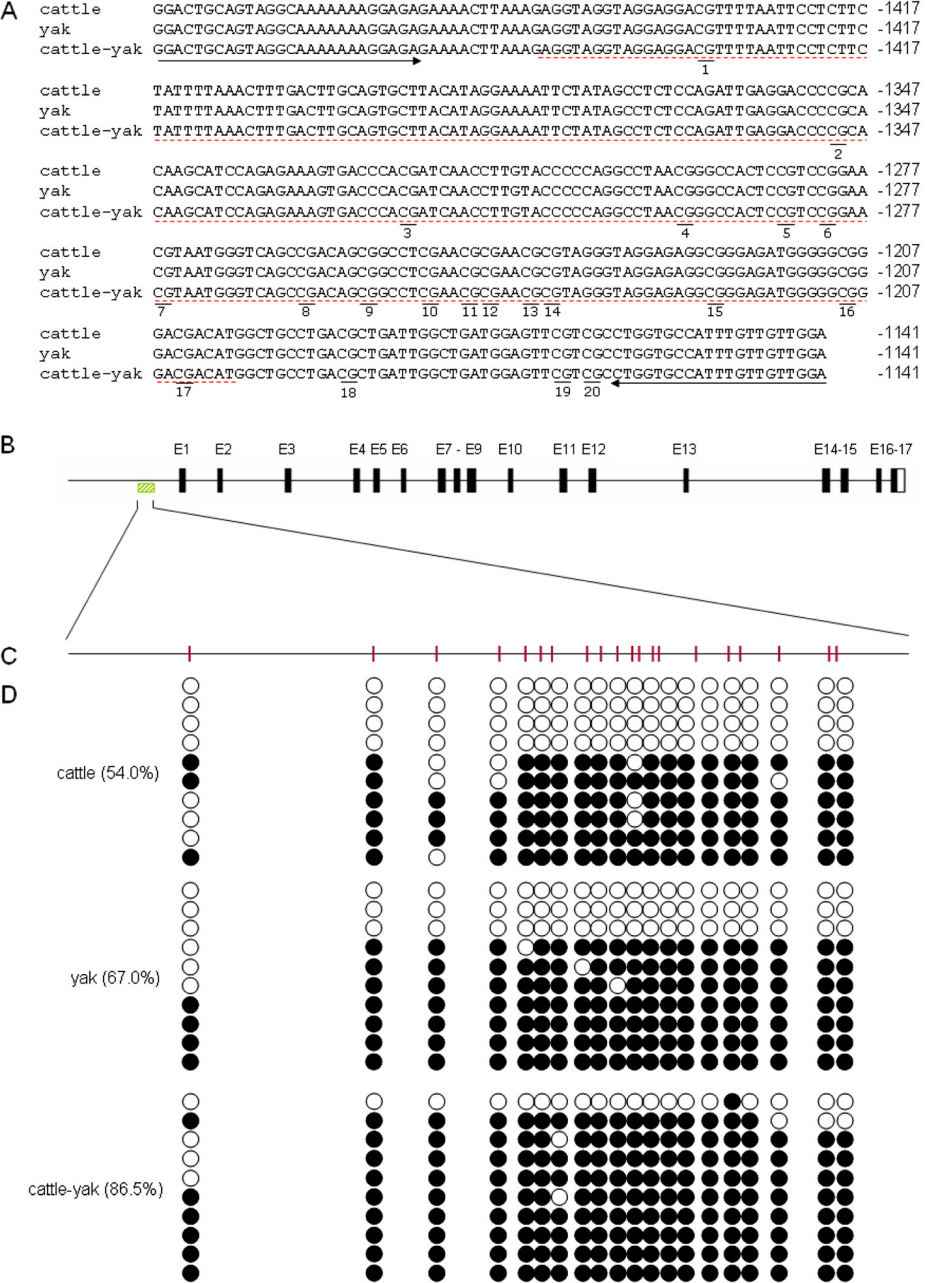
**Promoter methylation statues of *Bvh *gene in testis of cattle**, **yak and cattle**-**yak hybrid. A**. BSP sequence and distribution of CpG sites in *Bvh* promoter of cattle, yak and cattle-yak hybrid. Underline means CpG site. Arrow means primers for BSP. Dashed line indicate core promoter. Nucleotide numbering is relative to +1 at the initiating ATG codon. **B**. The genomic organization of the cattle *Bvh* gene. Numbered vertical lines indicate exons. Hatched boxes indicate BSP-amplyfied region. **C**. The CpG sites of BSP-amplified region. Vertical lines indicate 20 CpG site. **D**. Bisulfite sequencing results for cattle, yak and cattle-yak hybrid. Each line represents an individual bacterial clone which was sequenced. Black dots indicate methylated CpG sites, open circles indicate unmethylated CpG sites.

Based on the position of the core promoter region and CpG island, primers P8 was designed to amplify a sequence of 346 bp (−1486nt ~ −1141nt) for bisulfite sequencing PCR (BSP) analysis. PCR products were cloned and sequenced, and found to be consistent with the sequences of cattle, yaks and cattle-yak hybrids, which all included 20 CpG sites (Figure 6A-C). The methylation test results of the CpG sites of the *Bvh* promoter in the testis of cattle, yaks and cattle-yak hybrids are shown in Figure 6D. The degree of methylation (86.5%, 173/200) of the *Bvh* promoter region in the testes of cattle-yak hybrids was significantly higher than that of cattle (54.0%, 108/200) and yaks (67.0%, 134/200) (P < 0.01). In addition, among the 20 CpG sites, the degree of methylation of CpG_3_, CpG_4_, CpG_11_ and CpG_16_ sites (90%, 90%, 90% and 100%, respectively) in the testes of cattle-yak hybrids were significantly higher than that of cattle (30%, 40%, 30% and 50%, respectively) (P < 0.05), but were not significantly different to that of yaks (P > 0.05).

## Discussion and conclusion

### Molecular characterization of the Bvh gene

The present study identified and characterized the bovine vasa homolog (*Bvh*) gene from cattle, yaks and their interspecific hybrid cattle-yaks. Sequence analysis indicated that the Bvh protein is a true DEAD-box family member and vasa family member. Like other members of the DEAD-box family, Bvh also contains two recombinase A (RecA)-like helicase domains, Domain 1 (DEADc domain) and Domain 2 (HELICc domain) [2,23]. Within the helicase domains, there are at least eleven characteristic sequence motifs at conserved positions of Bvh, with seven conserved motifs (Q, I, Ia, Ib, GG, II and III) in Domain 1 and four motifs (IV, Iva, V and VI) in Domain 2, which is consistent with other mammals [2,6,19]. Previous investigations found that these conserved motifs are all involved with the function of Vasa, among which motifs Q, I and II are related to ATP binding, motif III related to hydrolysis, motif Ia, Ib, IV, and V related to RNA binding with RNA, and motif VI has a role in ATP activity and helicase activity [4,23]. Thus, the amino acid sequence, constitution, arrangement and location of functional domains and motifs of Bvh are highly similar to the Vasa proteins from other mammals, which suggests that the Bvh protein is a member of DEAD-box protein family with ATP-dependent RNA helicase activity, and plays an important role in bovine spermatogenesis [4].

### Alternative splicing of the Bvh gene

Alternative splicing (AS) is a major mechanism for the enhancement of transcriptome and proteome diversity, and plays important roles in development, physiology and in the pathology of various diseases, particularly in mammals [32]. Previous studies showed that at least 74% of human multi-exon genes are alternatively spliced [33]. Alternative splicing is a central tool of evolution that significantly increases the size of the transcriptome and generates functional specification. In the post-genomics era, AS has attracted the attention of researchers [34,35]. In this study, two splice variants were identified within the coding regions of *Bvh*: *Bvh*-*V4* and *Bvh*-*V45*. The alternative splice sites in *Bvh* are all located in the first five exons of the N-terminus (*Bvh*-*V4* lacks exon 4, and *Bvh*-*V45* lacks exon 4 and exon 5) and lead to amino acid deletions of the Bvh protein sequence.

Previous data showed that at least one expressed splice variant lacking an exon within the N-terminal region is present in other species, such as tammar and zebrafish [2,28]. In tammar and zebrafish, the shorter-splice variants all lack exon 4. In addition, screening of the GenBank database using BLAST showed that three splice variants exist in the human *Vasa* gene, compared with the full-length human *Vasa* cDNA (GenBank ID: NM_024415.2). Splice variant 1 (GenBank ID: NM_001166533.1) lacks 60 bp from exon 7 and exon 8, splice variant 2 (GenBank ID: NM_001142549.1) lacks 102 bp from exon 7 and exon 9, and the shortest, splice variant 3 (GenBank ID: NM_001166534.1), lacks 447 bp from exons 2–6 and exon9. The alternative splicing patterns of *Vasa* in chimpanzee and marmoset were exactly consistent with the human gene. The mouse *Mvh* transcript variant (GenBank ID: NM_010029.2) lacks 78 bp from exon 4. The lack of sequence conservation suggests that if the N-terminal region plays a specific role in *Vasa* regulation, it appears to be species specific [2]. The alternative splicing of *Bvh* occurred in the region encoding the N-terminal part of the protein, which does not contain functional domains and motifs; therefore, we speculated that protein isoforms Bvh-V4 and Bvh-V45 have similar functionality to Bvh.

### Expression of the Bvh gene

The *Vasa* gene is particularly expressed in mammalian germplasm cells, and is closely related to spermatogenesis and meiosis [19,20,36]. Previous studies found that many RNA metabolism-related processes, such as transcription, ribosome biogenesis, RNA splicing, editing, transferring and translation were regulated by *Vasa*[37,38]. Recently, studies observed that *Vasa* was involved in small RNA pathway, especially those closely related to mammalian spermatogenesis, such as the Piwi-interacting RNA (piRNA) [39,40]. In this study, we found that *Bvh* and two splice variants, *Bvh*-*V4* and *Bvh*-*V45*, are specifically expressed in the testes and ovary of adult cattle, which is consistent with the expression profile of *Vasa* in other mammals [19,41,42]. The results indicated that Bvh, Bvh-V4 and Bvh-V45 might, as in other mammals, make a significant contribution to the process of meiosis and *Bvh* might represent an important candidate gene that could influence bovine spermatogenesis. By real-time PCR, we found that the mRNA expression levels of *Bvh* in the testis of cattle and yaks with normal meiosis and spermatogenesis were significantly higher than that of cattle-yak hybrids with meiotic arrest (MA) and male sterility. The phenotype of MA and male sterility in cattle-yak hybrids [13] is consistent with the phenotype of *Mvh* gene knockout mice [10], suggesting that the mRNA levels of *Bvh* in the testicular tissue may be associated with the male sterility of cattle-yak hybrids. Ando et al. [43] found that transcription levels of *Vasa* in testicular tissue of successful testicular sperm extraction (TESE) patients with nonobstructive azoospermia (NOA) were higher than that of unsuccessful TESE groups, and suggested that measuring *Vasa* mRNA in testis could be a useful adjunct to conventional parameters for predicting sperm retrieval by micro-TESE in patients with NOA. The *Vasa* mRNA and protein levels were significantly decreased in patients with oligozoospermia: their mRNA level was only 1/5 of the normozoospermic men [44]. Thus, the low expression of *Vasa* is related to the pathogenesis of some subtypes of male infertility, and *Vasa* could be used as a molecular marker for the diagnosis of male infertility [44].

In cattle testes, the relative ratio for *Bvh*-*FL*: *Bvh*-*V4*: *Bvh*-*V45* was 2.2:1.6:1, and the differences in their expression levels were significant (P < 0.01 or P < 0.05). *Bvh*-*FL* and *Bvh*-*V4* were the most abundantly expressed isoforms in the testes of cattle with complete spermatogenesis. In the testes of cattle-yak hybrids with MA of spermatogenesis, transcript levels of the two splice variants were significantly decreased (P < 0.01). Collectively, these data suggest a major physiological role for *Bvh*-*V4* in bovine spermatogenesis between two splice variants.

### Promoter methylation status of Bvh in testes

During transcription, the regulation of TF binding sites and TF interaction can be achieved by epigenetic modifications of the DNA, including DNA methylation, one of the main genome epigenetic modifications [45,46]. To further study the mechanism of epigenetic regulation of *Bvh* expression in bovine testicular tissue, BSP was used to detect the methylation status of the *Bvh* promoter region in cattle, yaks and their interspecific hybrid cattle-yaks. The methylation level of the *Bvh* promoter region in the testicular tissue of cattle-yak hybrids (86.5%) was significantly higher than that of cattle (54.0%) and yaks (67.0%). These results indicated that the promoter region methylation of *Bvh* in testes is involved in transcriptional regulation, which was consistent with previous findings. The *Vasa* genes in humans and mice are regulated by the methylation state of tissue-specific differentially methylated regions (TDMRs). The methylation status of the CpG islands region in the promoter is related to the specific expression of *Vasa* and spermatogenesis, in which the *Vasa* promoter is hypomethylated in the testes but methylated in all other tissues that do not express *Vasa*[47]. A clinical study showed that spermatogenesis defects, such as idiopathic azoospermia or severe oligospermia, were also associated with a hypermethylated *Vasa* promoter in some individuals [48]. Lin et al. [49] reported that some germ cell-specific genes (e.g. Nanog, Pou5f1, and Zp1) in the marmoset and mouse testis showed different expression patterns and methylation patterns, but the expression patterns and methylation patterns of *Vasa* and some imprinted genes are conserved.

In addition, of the 20 CpG sites in the *Bvh* promoter, only CpG_3_, CpG_4_, CpG_11_ and CpG_16_ showed different methylation levels between cattle-yaks and their male parent (cattle). DNA methylation regulates gene transcription mainly through two mechanisms [50,51]. Firstly, gene transcription may be inhibited by blocking the binding between a TF and its binding sites in the promoter region. Secondly, the recognition and specific binding to DNA methylation sites by methyl-CpG-binding proteins (MBPs) influences TF binding, and thus inhibits transcription initiation. To explore the probably mechanism by which differentially methylated (DM) CpG sites affect the expression level of *Bvh*, the putative transcription factor binding patterns associated with the differentially methylated (DM) CpG sites were determined using the web tools TFSEARCH (with a threshold score of 85.0), MatInspector and Proscan. The results showed that CpG site CpG_3_ is located in the binding site for transcription factor GATA-1, while CpG_16_ is located in the binding site for transcription factors Sp1 and T-Ag. The transcription factor Sp1 is a member of the Sp family, whose zinc finger domain near the C-terminus can specifically recognize a GC Box on the DNA sequence. Sp TFs regulate transcription in multiple tissues [52]. Methylation of Sp1 binding sites in a promoter region tends to inhibit the transcription of the gene [53,54]. Therefore, we speculate that the hypermethylation of the Sp1 binding site (CpG_16_) in the *Bvh* promoter in the testicular tissues of cattle-yak hybrids is probably responsible for the lower expression of *Bvh*. Hypermethylation of Sp1 binding sites probably prevents Sp1 from binding to its binding sites by recruiting MBPs, thus inhibiting *Bvh* expression [53,55].

## Methods

### Sample collection and nucleic acid preparation

Healthy adult male cattle (n = 8), male yaks (n = 8), male cattle-yaks (n = 8) and female cattle (n = 2) were obtained from a slaughterhouse in Songpan in Sichuan Province, China, and slaughtered for tissue sampling of testis, ovary, epididymis, glandula accessoria, hypophysis, hypothalamus, heart, liver, spleen, kidney, lung and muscle. The samples were stored frozen in liquid nitrogen at −70°C for isolation of total tissue RNA. For the isolation of genomic DNA for polymerase chain reaction-restriction fragment length polymorphism (PCR-RFLP), blood samples from cattle (n = 78), yaks (n = 65) and cattle-yaks (n = 62) were collected from Gyamda, Lhasa, Tibet, China. All experiments were performed in accordance with the guidelines of the regional Animal Ethics Committee and were approved by the Institutional Animal Care and Use Committee of Nanjing Agricultural University.

Genomic DNA from blood and testis was extracted by a conventional phenol-chloroform extraction method. Total RNA was extracted using a Trizol kit (TaKaRa, Dalian, China). The reverse transcription reaction mixture for first-strand cDNA synthesis included 2 *μ*g of total RNA, 1 *μ*L of random primer, 200 U M-MLV reverse transcriptase (Promega, Madison, USA), 20 U RNAse inhibitor (Promega, Madison, USA), 5 *μ*L of × RT buffer (250 mM Tris–HCl, pH 8.3; 50 mM MgCl_2_; 250 mM KCl; 50 mmol/l DTT; 2.5 mM Spermidine) and 0.4 mM each of dNTP in a final volume of 25 *μ*L. The reverse transcription reaction was performed according to the manufacturer’s instructions. The genomic DNA and RT products were stored at −30°C.

### PCR amplification and clone sequencing

Primers P1-P4 were designed by Primer Premier v5.0 software based on the mRNA sequence of the cow vasa and cow *β*-*actin* gene. Primers P8 for BSP were designed based on the genomic sequence of the cow vasa gene (contained in the genomic scaffold sequence accession no. NW_003104511) using Methyl Primer Express v1.0 software (Applied Biosystems, Foster City, CA, USA). The primer sequences and PCR conditions are shown in Table 1.

All the PCR reactions were performed in 10 *μ*L containing 0.5 *μ*L of RT products (or genomic DNA), 1 U Ex Taq DNA polymerase (TaKaRa), 1 *μ*L of 10× PCR Buffer, 0.25 mM dNTP, 1.25 mM MgCl_2_ and 10 pM of each primer. The following cycling conditions were used: 95°C for 5 min; 35 cycles of 95°C for 45 s, annealing for 30 s and 72°C for 5 min. PCR products were fractionated on a 1.5% agarose gel and purified using a DNA Purification Kit (Axygen, Union City, CA, USA). The ligation product of the target gene and vector pMD18-T (TaKaRa) was transferred into *Escherichia coli* strain JM109. Positive clones were picked and plasmid DNA was extracted with a Plasmid DNA Extraction Kit (Axygen), and sequenced by Shanghai Invitrogen Co. (Shanghai, China).

### Bioinformatic analysis

Editing and translation of the nucleotide sequence was executed using DNAStar 5.22 software (DNASTAR, Madison, WI, USA). The BLAST server (http://www.ncbi.nlm.nih.gov/BLAST/) was used to search for homologous sequences. Nucleotide and amino acid sequences alignment was performed using Clustal W (http://www.ebi.ac.uk). Motif analysis was performed using the online programs MotifScan (http://myhits.isb-sib.ch/cgi-bin/motif_scan) and NetPhosk 1.0 (http://www.cbs.dtu.dk/services/NetPhosk/). Genomic organization, chromosomal locations and chromosomal synteny analysis were investigated by comparing the cDNA and corresponding genomic sequence (http://genome.ucsc.edu/).

The polymorphic sites, numbers of transitions (Ts), numbers of transversions (Tv), and transition/transversions (Ts/Tv) were analyzed using MEGA5.1 software [56]. The nucleotide diversity (*π*), nonsynonymous substitutions (d_N_), number of synonymous substitutions (d_S_), and d_N_/d_S_ were determined by the DNASP 5.0 program [57]. Phylogenetic trees were constructed using the maximum parsimony method in the MEGA5.1 software by selecting the Kimura 2-parameter model, and bootstrap percentage values were obtained by a bootstrap replications test (1000 replications). The putative promoter region of *Bvh* was predicted using Proscan software (http://www-bimas.cit.nih.gov/molbio/proscan/). The CpG Island Searcher program (http://ccnt.hsc.usc.edu/cpgislands2/cpg.aspx) was used to identify CpG islands. Putative TF binding sites were predicted using the web tools Proscan, TFSEARCH (http://www.cbrc.jp/research/db/TFSEARCH.html) and MatInspector (http://www.genomatix.de/).

### PCR-RFLP analysis

Primers P5 for PCR-RFLP were designed based on the cDNA sequence of the *Bvh* gene, *Bvh* genomic sequence and polymorphic sites (Table 1). The PCR products were digested with 8 U of restriction enzyme *Nde* I (NEB, UK) overnight at 65°C. The digestion products were then resolved on agarose gels stained with ethidium bromide.

### Real-time PCR

The levels messenger RNA of *Bvh* and its splice variants in testis tissues of cattle, yaks and cattle-yaks were assessed by real-time PCR using a fluorescence temperature cycler (MJ Research, Waltham, MA, USA), and normalized to the level of mRNA of the housekeeping gene *β*-actin to compensate for variations in the amounts of input RNA. Real-time PCR was performed according to the *ΔΔ*Ct method described by Livak and Schmittgen [58].

### Methylation analysis

Bisulfite modification of testicular genomic DNA was performed using the MethylCode™ Bisulfite Conversion Kit (Invitrogen, Shanghai, China). The PCR products were cloned into vector TA pCR2.1 (Invitrogen) and transformed into competent E. coli Top10 cells (Tiangen, China). Blue-white selection and PCR were used to verify the cloning step. The Shanghai Invitrogen Co. (Shanghai, China) purified the plasmids and sequenced the inserts. The positions and percentages of methylated cytosine residues for all non-CpGs were then determined by aligning the sequenced results with the bisulfite-modified DNA sequences converted from Methyl Primer Express 1.0 software.

### Statistical analysis

Statistical analysis was performed using SPSS v16.0 For Windows. Significant differences were set at P < 0.05.

## Abbreviations

Bvh: Bovine vasa homology; RFLP: Restriction Fragment Length Polymorphism; SV: Splice Variant; MBPs: Methyl-CpG-Binding Proteins.

## Competing interests

The authors declare that they have no competing interests.

## Authors’ contributions

HL and YZ performed the study, prepared the manuscript and analysed the data. HL and YL analysed the data and drafted the paper. QL designed the study, analysed the data and prepared the manuscript. All authors read and approved the final manuscript.
